# Non-motor symptoms associated with progressive loss of dopaminergic neurons in a mouse model of Parkinson’s disease

**DOI:** 10.3389/fnins.2024.1375265

**Published:** 2024-04-30

**Authors:** Anna Radlicka-Borysewska, Judyta Jabłońska, Michał Lenarczyk, Łukasz Szumiec, Zofia Harda, Monika Bagińska, Justyna Barut, Joanna Pera, Grzegorz Kreiner, Daniel K. Wójcik, Jan Rodriguez Parkitna

**Affiliations:** ^1^Department of Molecular Neuropharmacology, Maj Institute of Pharmacology of the Polish Academy of Sciences, Kraków, Poland; ^2^Faculty of Management and Social Communication, Institute of Applied Psychology, Jagiellonian University, Kraków, Poland; ^3^Department of Brain Biochemistry, Maj Institute of Pharmacology of the Polish Academy of Sciences, Kraków, Poland; ^4^Department of Neurology, Jagiellonian University Medical College, Kraków, Poland; ^5^Laboratory of Neuroinformatics, Nencki Institute of Experimental Biology of the Polish Academy of Sciences, Warsaw, Poland

**Keywords:** Parkinson’s disease, dopamine, mouse, TIF-IA^DATCreERT2^, behavioral tests, non-motor symptoms, executive function

## Abstract

Parkinson’s disease (PD) is characterized by three main motor symptoms: bradykinesia, rigidity and tremor. PD is also associated with diverse non-motor symptoms that may develop in parallel or precede motor dysfunctions, ranging from autonomic system dysfunctions and impaired sensory perception to cognitive deficits and depression. Here, we examine the role of the progressive loss of dopaminergic transmission in behaviors related to the non-motor symptoms of PD in a mouse model of the disease (the TIF-IA^DATCreERT2^ strain). We found that in the period from 5 to 12 weeks after the induction of a gradual loss of dopaminergic neurons, mild motor symptoms became detectable, including changes in the distance between paws while standing as well as the swing speed and step sequence. Male mutant mice showed no apparent changes in olfactory acuity, no anhedonia-like behaviors, and normal learning in an instrumental task; however, a pronounced increase in the number of operant responses performed was noted. Similarly, female mice with progressive dopaminergic neuron degeneration showed normal learning in the probabilistic reversal learning task and no loss of sweet-taste preference, but again, a robustly higher number of choices were performed in the task. In both males and females, the higher number of instrumental responses did not affect the accuracy or the fraction of rewarded responses. Taken together, these data reveal discrete, dopamine-dependent non-motor symptoms that emerge in the early stages of dopaminergic neuron degeneration.

## Introduction

The primary motor symptoms of Parkinson’s disease (PD) are caused by progressive degeneration of dopaminergic neuron projections from the ventral midbrain to the basal ganglia and the resulting imbalance in the activity of the direct and indirect striatal pathways (Hornykiewicz, 1998; McGregor and Nelson, 2019). PD is also associated with a variety of non-motor symptoms that include impaired sensory perception (Boecker et al., 1999; Cury et al., 2016; Weil et al., 2016; Honma et al., 2018), autonomic dysfunction (Jain and Goldstein, 2012; Yeo et al., 2012; Fasano et al., 2015; Chen et al., 2020), cognitive impairments, dementia and depression (Aarsland et al., 2010, 2012; Aarsland and Kurz, 2010; Schapira et al., 2017). The wide diversity of the non-motor symptoms is linked to the stages of development of PD, with neuron degeneration possibly initially affecting the olfactory bulb in the brain and/or the enteric nervous system before advancing onto the sympathetic system, then spreading through the hindbrain, causing degeneration of noradrenergic and dopaminergic neurons, and eventually reaching the forebrain in the late stages (Braak et al., 2006; Hawkes et al., 2007; Schapira et al., 2017; Beach et al., 2021). Accordingly, the loss of olfactory acuity may precede motor symptoms and may even serve as an early indicator for diagnosis (Bohnen et al., 2008; Doty, 2012; Durcan et al., 2019). In the late stages of the disease, the majority of PD patients develop dementia (Aarsland and Kurz, 2010). This dementia is primarily related to an increasing load of Lewy bodies in the neocortical areas and degeneration of cholinergic neurons within the nucleus basalis (Halliday et al., 2014).

Parkinson’s disease (PD) is a multisystem disease (Jellinger, 2012) that affects multiple neurotransmitter systems, including the dopaminergic, noradrenergic, serotonergic (Buddhala et al., 2015) and cholinergic (Müller and Bohnen, 2013; Perez-Lloret and Barrantes, 2016) systems. However, the extent to which the loss of dopamine contributes to specific non-motor symptoms is varied and in many cases remains controversial. For instance, while cognitive deficits in the later stages of PD were primarily attributed to cholinergic degeneration and thus resemble the symptoms of dementia associated with Alzheimer’s disease, an impairment of executive functions is reportedly specific to PD and includes deficits in attention, impaired problem solving and action sequencing (Emre, 2003). These symptoms are also reported in PD without dementia (Fengler et al., 2017) and could be plausibly explained by impaired dopamine signaling in the basal ganglia. Executive dysfunctions, already present in a considerable fraction of newly diagnosed patients (Muslimović et al., 2005), may stem from dopamine deficits that affect fronto-striatal loops, while PD dementia that emerges in later PD stages could be caused by the impairment of other neurotransmitter signaling (Kehagia et al., 2010). Although neuroimaging evidence points to the dopaminergic origins of executive dysfunctions in PD (Han et al., 2021), the correlation of their severity with cardinal motor symptoms, also dependent on dopamine signaling, gives mixed results between the studies (Campos-Sousa et al., 2010; Murakami et al., 2013; Schneider et al., 2015). Moreover, the incidence of depression was significantly increased in the prodromal phase of PD and was correlated with altered hyperechogenicity of the substantia nigra (Walter et al., 2007), which again points to altered dopaminergic transmission as a potential cause. Nevertheless, in the cases of executive impairments or depression, the degeneration of the locus coeruleus noradrenergic neurons could be argued to be an equally plausible cause, as could the impact of disrupted cholinergic transmission. Finally, limited correlations exist in the development of motor and non-motor symptoms (Ravan et al., 2015; Mu et al., 2017; Rodriguez-Sanchez et al., 2021), and attempts to classify PD subtypes on the basis of the cooccurrence of both types of symptoms remain inconclusive (Qian and Huang, 2019).

Therefore, to isolate the contribution of dopaminergic neurons in the development of non-motor symptoms of PD, the model of choice is genetically modified mice with an inducible loss of dopaminergic neurons by the use of the Cre-loxP system that targets functionally essential genes in DAT-expressing neurons, such as in MitoPark (Ekstrand et al., 2007) and TIF-IA^DATCreERT2^ strains (Rieker et al., 2011). The use of these mice allows us to study the Parkinsonian phenotype that develops relatively slowly (compared to neurotoxic models) and is selectively caused by dopaminergic dysfunction. Although these models have, arguably, limited construct validity (i.e., the underlying cause of the loss of neurons is not the same as in PD), they were shown to accurately recapitulate motor impairment (Ekstrand et al., 2007; Rieker et al., 2011; Gellhaar et al., 2015; Kreiner et al., 2019), selected non-motor symptoms of PD (Li et al., 2013) and exhibit at least some cell loss mechanisms similar to that of PD, i.e., mitochondrial dysfunction or nucleolar disruption. Therefore, they proved valuable in investigating the consequences of the loss of dopaminergic neurons and testing new potential treatments for PD. Here, we use the TIF-IA^DATCreERT2^ strain to assess the development of motor and non-motor symptoms in the early and middle stages of dopaminergic neuron degeneration. A major advantage of this model is that it was previously extensively characterized with regard to the progression of the degeneration of dopaminergic neurons. The loss of the TIF-IA factor in adult mice causes nucleolar disruption and appreciable loss of dopaminergic neurons starting around 7 weeks after mutation induction in the substantia nigra pars compacta (SNpc), then reaching ∼90% loss in the SNpc and ∼70% degeneration in the ventral tegmental area (VTA) 21 weeks after induction of the mutation (Rieker et al., 2011; Kreiner et al., 2019). In this study we focused on the emergence of changes in gait together with symptoms related to impaired olfaction, anhedonia-like behavior and cognitive performance in the early stages of dopaminergic degeneration.

## Materials and methods

### Animals

Mice were housed at the animal facility of the Maj Institute of Pharmacology of the Polish Academy of Sciences under a 12/12 h light/dark cycle at 22 ± 2°C and 55 ± 10% air humidity. Males were housed in groups of 2–4 siblings per cage, except for saccharin preference test sessions, during which they were housed individually for periods of 24 h. Females were initially housed 2–4 per cage and were later moved to two IntelliCages, 11 females in each. Animals had *ad libitum* access to food and water, except for male mice assessed for olfactory acuity (buried food task), which were deprived of food access for 24 h prior to the test.

Male and female TIF-IA^DATCreERT2^ [Tg/0; flox/flox] mice were generated as described previously (Rieker et al., 2011; Kreiner et al., 2019). Control animals lacked the CreERT2 transgene ([0/0; flox/flox] genotype). *Tif1a* inactivation in dopamine transporter (DAT)-expressing cells was induced in 9- to 11-week-old male and female mice by tamoxifen treatment (Sigma, Germany); specifically, 2 mg dissolved in 100 μl of sunflower oil was injected once daily i.p., for 5 consecutive days. Control mice also received tamoxifen treatment. All mice were allowed to rest for 3 weeks before testing started. TIF-IA is an essential transcription factor for RNA polymerase I (Miller et al., 2001; Yuan et al., 2002) and its loss first causes disruption of the nucleolus and over time cell death through apoptosis (Yuan et al., 2005). The progression of the loss of dopaminergic neurons in TIF-IA^DATCreERT2^ mice was characterized in two previous reports (Rieker et al., 2011; Kreiner et al., 2019). Experimental procedures were performed on 22 males (10 mutants and 12 controls) and 22 females (9 mutants and 13 controls), including 1 control female that was excluded from the probabilistic reversal learning test in the early adaptation phase.

Female animals were tested in an IntelliCage, while males were assessed in a battery of behavioral tests. The rationale was that due to lower aggression levels females are better suited for automatic testing without experimenter supervision, and conversely, the scent of females could affect behavior in operant boxes and other tests, and those were performed only on male mice. Gait analysis was performed on both sexes.

All procedures were conducted in accordance with the ARRIVE guidelines, Polish law and European Commission regulations concerning the care and use of laboratory animals (Directive 2010/63/UE, European Convention for the Protection of Vertebrate Animals Used for Experimental and other Scientific Purposes ETS No.123, and Polish Law Dz.U. 2015 poz. 266). All experimental procedures were approved by the II Local Institutional Animal Care and Use Committee in Kraków (permits no. 28/2019, 197/2019 and 198/2019).

### Behavioral procedures

**FIGURE 1:**
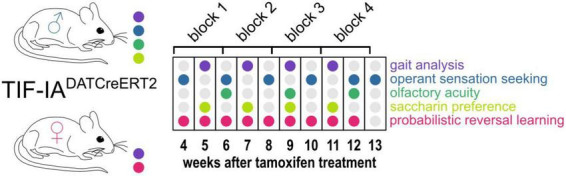
Experimental design. The diagram shows the schedule of gait testing (Catwalk, violet dots) and non-motor behaviors tested (all remaining colors, as indicated on the left) in male and female TIF-IA^DATCreERT2^ mice and controls. 16-day-long blocks used for performing correlation analyses are also shown at the top in relation to weeks after tamoxifen treatment.

Male and female mice underwent regular testing for gait parameters and behaviors related to non-motor PD symptoms starting on the 4th week after tamoxifen injection. A schematic representation of the experiment is shown in Figure 1. In addition to gait analysis, male mice were tested for operant sensation seeking, olfactory acuity and saccharin preference. Female mice remained in an IntelliCage apparatus throughout the procedure (except for the gait recording sessions) and were tested for probabilistic reversal learning. The body weights of all animals were also monitored.

#### Gait

All mice were tested for gait on the 5th, 7th, 9th, and 11th week after tamoxifen injection using the CatWalk XT system v10.6 (Noldus, Netherlands). The tests were performed during the light phase. Briefly, mice were placed individually at either of the two ends of a tunnel covering a side-lit glass platform and were allowed to move freely in the tunnel. A camera placed under the platform recorded the body movement and paw placement, and the recordings were validated by the software. A total of 2–6 runs of each mouse per test session were used for statistical analyses. Recordings that did not include at least 1 complete pattern detected were excluded from the analysis.

#### Operant sensation seeking

The operant sensation seeking test was performed as described previously (Olsen and Winder, 2009; Rodriguez Parkitna et al., 2013; Sikora et al., 2019). The task models the sensation-seeking trait, and was previously shown to be affected by dopamine and excitatory signaling (Olsen and Winder, 2009; Sikora et al., 2019) and also the endogenous opioid system. A major advantage of the task in the context of TIF-IA^DATCreERT2^ mice is that it allows to observe operant learning without the necessity of food or water deprivation. Male mice were placed individually in operant conditioning chambers ENV-307W (Med Associates, USA). During the 1-hour sessions, the animals were able to freely explore the cage and perform active and inactive operant responses by placing their snouts in one of the two holes located ∼2 cm above the grid floor. The active operant response resulted in the presentation of a blinking light with a randomized duration of 2, 4, 6 or 8 s, a frequency of 0.625, 1.25, 2.5, or 5 Hz and a 2.9 kHz 65 dB monotone beep (ENV-323AW Sonalert, Med Associates). The assignment of active and inactive operant holes was fixed for each mouse throughout the experiment. The number of active and inactive operant responses was recorded by the software.

#### Olfactory acuity

Olfactory acuity was assessed in the buried food test as described by Yang and Crawley (2009). One to three days before each test, mice received vegetable-flavored crackers in their home cages (“Sunbites” brand, Frito Lay Poland). All food was removed from the home cage on the day prior to the test. Olfactory acuity was assessed by placing the mice individually in an opaque cage (interior dimensions: 53 cm × 32 cm × 19 cm) filled with a 3-cm layer of aspen bedding. First, a mouse was able to explore the cage freely, and after acclimatization, it was briefly transferred to a separate cage while a cracker (approx. 2 g) was buried beneath the bedding in one of the corners. The mouse was then reintroduced to the opposite corner of the cage. The observer recorded the delay before the mouse started digging in the correct corner and the time to retrieve the cracker (i.e., digging out or taking a bite). The location of the cracker was randomized and changed after each completed trial.

#### Saccharin preference

Saccharin preference was measured as described previously (Skupio et al., 2015) with minor modifications. Males were placed in individual small cages (26 cm × 20 cm × 135 cm) for 24 hours and were able to drink tap water or a 0.1% (w/v) saccharin solution (Sigma Aldrich, Germany) from measuring cylinders. Fluid consumption was assessed based on the change in liquid volume. The position of the cylinders was switched between consecutive tests. Changes in volume ≥ 20 ml were assumed to result from leaks and were excluded from analysis.

#### Probabilistic reversal learning

The probabilistic reversal learning task was performed as described previously (Jabłońska et al., 2021). Briefly, female TIF-IA^DATCreERT2^ mice were implanted subcutaneously with PICO UNO radio-frequency identification (RFID) transponders 4 weeks after mutation induction and placed in groups of 11 in two automated IntelliCage systems (TSE Systems, Germany). Each group included both genotypes. The rationale for using only female mice in this task is their lower level of aggressive behaviors. Animals in the IntelliCage have minimal contact with the experimenter (when they were tested for catwalk and during biweekly bedding changes), which limits the ability to control for fighting marks or wounds. The cages recorded all entries of an animal into a corner and counted the number of licks on each of the bottles placed in the corners. Each corner was fitted with two 250-ml bottles, accessible through ports with access controlled by sliding doors. Food was available *ad libitum* throughout the procedure. At the start of the procedure, mice were introduced into the IntelliCage and allowed free access to all the drinking bottles (i.e., the sliding doors were in the open position). Next, bottles in two of the corners were replaced with 0.1% (w/v) saccharin solution, and a restriction was placed on access: the doors blocking the access to bottles opened for 10 s after the animal was detected in the corner, with a 0.5 s delay in the case of water bottles and a 2 s delay for saccharin bottles. The bottles filled with saccharin solution could be accessed by mice in 90% of visits (in a random order, with fixed probability of access), while water remained fully accessible. The position of the saccharin and water bottles was changed every 48 hours to avoid corner preference. After 3 position changes, the probabilistic reversal phase started. The position of the corners with saccharin bottles was fixed, but access to the bottles was granted with 90% probability in one of the corners and 30% in the other. The probabilities were reversed every 48 hours, and the experiment continued until the end of 12 weeks after the mutation was induced. The procedure was interrupted several times, e.g., for brief periods of several hours required for gait testing, weight measurements, cage cleaning and maintenance. Periods of time with interruption of correct system operation were excluded from the analysis (a complete list is provided in the script used for the statistical analysis).

### Immunofluorescence staining

On the 123rd day (18th week) after tamoxifen treatment, the animals were killed by cervical dislocation to confirm mutation-induced complete loss of dopaminergic neurons. Their brains were extracted and fixed in 4% paraformaldehyde (Roth, Germany) solution at 4°C for two days, and the fixative was then changed to 0.4% paraformaldehyde. After 55 days, the brains were stored in phosphate-buffered saline solution (PBS; Serva, Germany) with 0.05% thimerosal (Sigma). The brains were sectioned on a Leica VT1200 (Leica, Germany) vibratome into 40-μm coronal sections, including the olfactory bulbs [bregma 4.28 to 3.92 mm according to the mouse brain atlas by Paxinos and Franklin (2001)], forebrain (bregma 1.54 to 0.14 mm) and midbrain (bregma −2.92 to −3.16 mm). The sections were collected in 48-well plates filled with 150 μl of PBS and refrigerated until staining.

The staining procedure was performed in accordance with a previously published protocol (Mustafa et al., 2021). After washing in PBS with 0.2% Triton X-100 (Amresco, USA), the sections were blocked for 30 minutes in 5% normal pig serum (Vector Laboratories, USA) in PBS with 0.2%-Triton X-100 and then incubated overnight at 4°C with anti-tyrosine hydroxylase (TH) primary antibodies (1:500; Anti-Tyrosine Hydroxylase Antibody, catalog no. AB1542; Sigma-Aldrich, USA) in the same blocking solution. The sections were then rinsed with PBS-Triton X-100 solution and incubated for 1 hour with the secondary antibody (1:100; rabbit anti-sheep IgG antibody (H + L), fluorescein, cat. no. FI-6000; Vector Laboratories), rinsed again with PBS and mounted with VECTASHIELD^®^ HardSet™ Antifade Mounting Medium with DAPI (Vector Laboratories). Images were captured with a Leica TCS SP8 X confocal laser-scanning microscope (Leica Microsystems, Germany) using HC PL FLUOTAR 10× (0.30) and 40× (0.60) objectives and Leica Application Suite X software (Leica Microsystems).

Additionally, to confirm that current procedure yields consistent results with the already published characterization of the strain, loss of dopaminergic neurons was assessed in a separate cohort of TIF-IA^DATCreERT2^ male mice at 14 weeks after tamoxifen treatment (the mutation effects were induced at the age of 12 weeks). The brains were extracted after killing the animals by cervical dislocation and fixed in 4% paraformaldehyde overnight at 4°C. Alcohol dehydrated tissue was then embedded in paraffin (Leica) and sectioned (7 μm) on a RM45 microtome (Leica). Adjacent coronal sections from corresponding regions of the substantia nigra/ventral tegmental area of the control and TIF-IA^DATCreERT2^ mice were incubated overnight at 4°C with the same anti-TH primary antibodies as mentioned above. Antigen-bound primary antibodies were visualized with appropriate secondary antibodies (Alexa 488, anti-sheep, cat. no. A-11015; Invitrogen). Stained sections were acquired and analyzed under a fluorescence TCS SP8 WLL microscope (Leica). Quantification of TH-positive neurons was performed manually by counting all TH+ cells on 3 adjacent sections from 3 animals (bregma: −3. 52; −3.64; −3.8 mm) of each genotype in a single-blind experiment.

### Statistical analysis

CatWalk data were first reduced to exclude variables without variance, missing measurements, and variables that were directly influenced by animals’ speed, since they may have been influenced by experimenters. Next, the corresponding parameters collected from the left and right paws were correlated and averaged, and parameters that had weak correlation (Pearson correlation coefficient *R* < 0.5) were excluded from further analyses, as they introduced noise into the analysis. To extract gait measures of interest that were potentially affected by partial loss of dopamine neurons, a logistic regression model, glm (formula = genotype ∼ sex + days + parameter, family = binomial), was then applied. A stepwise variable selection algorithm was used to select variables relevant for the prediction of genotype from data, starting from a null model comprising only date and sex as predictors.

The parameters included in the final regression model as a result of the selection algorithm were further analyzed in R v4.0.4. Both CatWalk data and measurements collected from tests evaluating non-motor functions and body weight were individually subjected to a mixed two-way analysis of variance (ANOVA) to determine the effects of mutation and the time elapsed and their interaction. Animals that lacked data in at least one time point for a variable were excluded from the analysis of that particular parameter. Additionally, Bonferroni-adjusted *t*-tests were carried out to assess differences between the groups at each time point as *post hoc* analyses. The analyses for males and females were carried out separately.

The correlation analysis of motor and non-motor function measurements was performed as follows: starting from the 23rd day after the last day of tamoxifen treatment, time points were grouped into four 16-day-long blocks (Figure 1). Blocks 2, 3 and 4, i.e., the results collected from the 39th day after tamoxifen treatment onward, were considered in the analysis. Olfactory acuity results and female weight were excluded from the correlation analysis due to missing data. An |R| value of ≥ 0.4 was significant and corresponded to a *P*-value of < 0.05 (Bonferroni adjusted for multiple parallel correlations).

## Results

### Loss of dopaminergic neurons in TIF-IA^DATCreERT2^ mice

**FIGURE 2:**
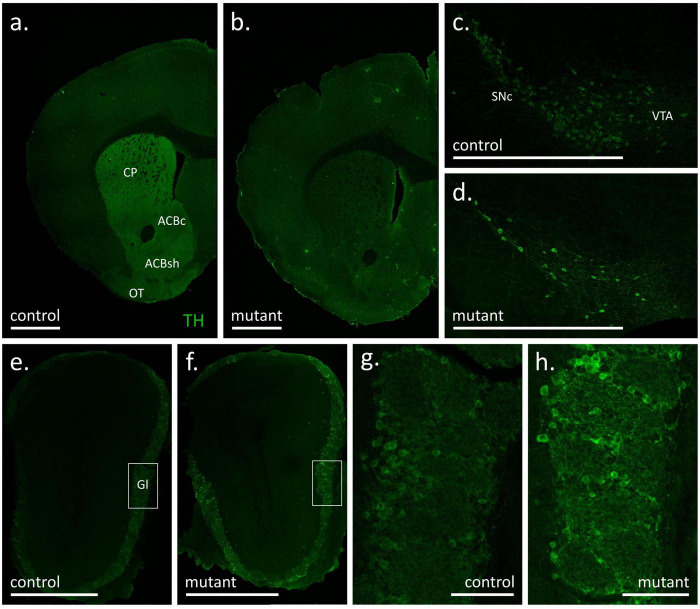
Tyrosine hydroxylase (TH) immunofluorescence signal in coronal sections of the forebrain **(a,b)**, substantia nigra with ventral tegmental area **(c,d)** and olfactory bulbs **(e–h)** of control **(a,c,e,g)** and mutant **(b,d,f,h)** mice at 18 weeks after tamoxifen treatment. White bars correspond to 1 mm **(a–f)** or 100 μm **(g,h)**. ACBc, nucleus accumbens, core; ACBs, nucleus accumbens, shell; CP, caudate putamen; Gl, glomerular layer of the olfactory bulb; OT, olfactory tubercle; SNc, substantia nigra pars compacta; VTA, ventral tegmental area.

The loss of dopamine-transporter (DAT) expressing neurons in the TIF-IA^DATCreERT2^ after induction of the mutation with tamoxifen was characterized previously (Rieker et al., 2011; Kreiner et al., 2019). To ensure consistency with previous results, we have used TH-immunofluorescence in brain sections derived from the cohorts of animals used for behavioral testing after the procedures were completed and also a separate cohort that was killed at a time point of 14 weeks, which corresponds to the late stage of behavioral testing (Figure 2 and Supplementary Figure 2).

In experimental animals killed 18 weeks after mutation induction robust TH staining was observed in the dorsal striatum, the nucleus accumbens and the olfactory tubercle of control animals, i.e., structures with the most extensive dopaminergic innervation (Ikemoto, 2010), while the fluorescence signal was notably weaker in the adjacent cortical areas (Figure 2a). Conversely, immunofluorescence in the corresponding striatal areas in a representative section from a TIF-IA^DATCreERT2^ mouse was weaker or absent and comparable to the signal observed in the dorsal cortical areas (Figure 2b). Accordingly, a difference in TH staining was also observed at the level of the midbrain, where the number of TH-positive cell bodies was appreciably larger in control animals (representative example in Figure 2c) than in mutants (Figure 2d). In line with a previous report (Papathanou et al., 2019), the DATCreERT2-driven mutation had no appreciable effects in the main olfactory bulb, where a similar intensity of fluorescence was observed in control (Figure 2e) and mutant (Figure 2f) animals. In both cases, intense TH immunofluorescence was located within the external, glomerular layer, which contains the dopamine- and GABA-releasing interneurons of area A16 (Björklund and Dunnett, 2007; Pignatelli and Belluzzi, 2017). As shown in the close-up Figures 2g, h, the TH signal was present both in cell bodies and neurites in the glomerular layer, again with no appreciable differences between mutant and control animals. Thus, at 18 weeks after tamoxifen administration, the mutation caused an extensive loss of TH-positive cells in the ventral midbrain but had no observable effect on the abundance of putative dopaminergic cells in the olfactory bulb. The extent of the mutation within the striatal and midbrain areas was consistent with the originally reported analysis, and also served as an additional validation of correct induction of the mutation (Rieker et al., 2011).

In order to ensure that the loss of dopaminergic neurons in TIF-IA^DATCreERT2^ animals was consistent with previous reports (Rieker et al., 2011; Kreiner et al., 2019), a separate cohort of animals was killed 14 weeks after tamoxifen treatment. As shown in Supplementary Figure 1, a difference in the number of TH-positive cells between controls and mutants was apparent throughout in anterior, middle and posterior parts of the midbrain. In line with previous reports, the loss of TH-positive cells in the SNpc was over 90% (Supplementary Figure 2a), and approximately 65% in the VTA (Supplementary Figure 2b).

**FIGURE 3:**
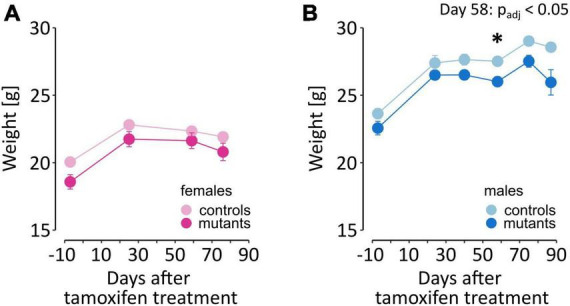
Weight of TIF-IA^DATCreERT2^ and control mice. **(A,B)** The graphs show the weight of the experimental animals immediately before tamoxifen treatment (–7 days time point) and throughout the testing procedure. Each point represents the group mean, and error bars are SEM. Female mice are shown in shades of pink, and male mice are shown in blue. Darker hues are TIF-IA^DATCreERT2^ mice, and lighter hues represent the controls. Star (*) indicate significant difference mutants vs. controls at the indicated time point, *t*-tests with Bonferroni correction for multiple tests, *p* < 0.05.

An additional essential control of the effects of the mutation are the changes in weight. Dopamine signaling is necessary for eating-oriented behavior. In humans, changes in weight were reported in PD patients in the premotor phase of the disease (Kistner et al., 2014). In mice, a complete loss of dopamine production is fatal, as animals do not consume sufficient amounts of food for sustenance (Szczypka et al., 1999). Here, we found that TIF-IA^DATCreERT2^ males were on average 5% lighter than the controls and that this trend was observable (but not significant) even before the induction of the mutation [*F*_(1,18)_ = 7.026, *P* = 0.0163; Figure 3A; a full list of all ANOVAs for the performed tests and measurements is included in Supplementary Table 1]. The difference in weight increased after the mutation was induced, as evidenced by the significant interaction of the genotype and time elapsed from tamoxifen administration [*F*_(5,90)_ = 2.56, *P* = 0.0326]. However, the weight continued to significantly increase over time in all animals irrespective of genotype [*F*_(5,90)_ = 87.08, *P* < 0.0001]. In females (Figure 3B), body weight also increased over time [*F*_(3,57)_ = 128.663, *P* < 0.0001], but the effects of the mutation [*F*_(1,19)_ = 3.32, *P* = 0.0842] and interaction [*F*_(3,57)_ = 1.773, *P* = 0.163] were not significant. Therefore, although all mice gained weight throughout the experiment, a relatively mild effect of the loss of dopamine cells was observed in male mutants.

### Effects of the mutation on motor function

To assess the effect of the progressive loss of dopaminergic neurons on motor function in TIF-IA^DATCreERT2^ mice, we conducted gait analysis using the CatWalk system (a list of gait characteristics and parameters is provided in Table 1). Animals were tested 4 times in 2-week intervals, starting on the 5th week after the induction of the mutation. Over 200 raw parameters and derived measures were generated from each session (see Supplementary Table 2). Two-step data reduction was performed. First, measures that were not recorded or were confounded by the specific setup of the experiment were removed. Next, a total of 31 parameters with only zeroes or no values scored were discarded. Additionally, we removed 7 parameters directly related to animals’ speed because the experimenter had to intervene at the start of a considerable fraction of trials. Second, correlations between left and right paw measures were assessed for the front and hind limbs, respectively (see Supplementary Table 3 for summary). Measures with Pearson *R* < 0.5 were excluded as having excessive variance, and the remaining parameters were averaged between the paws. A total of 62 parameters remained after data reduction (Supplementary Table 4) and were used to construct a logistic regression model with the fewest parameters that achieved the highest accuracy in predicting the genotype. The optimal model included 15 gait parameters and 3 days × parameter interactions (AIC = 700.3788, cross-validated accuracy = 0.688, Supplementary Table 5), primarily related to the hind paw print size, paw placement coordination and timing, and cadence. Thus, these 15 gait parameters were indicated to be most closely related to the animals’ genotype.

**TABLE 1:** Description of selected gait characteristics and parameters measured by the CatWalk system in this study.

Gait characteristic	Description	Example of CatWalk parameters [units]
Stand or Stance	A phase of gait during which a paw is in contact with glass walkway/walking surface.	Stand [s]
Swing	A phase of gait during which a paw is not touching the walkway.	Swing speed [cm/s]
Step cycle	Time between two consecutive initial contacts of the same paw and the walkway; it equals the sum of stand and swing times during a single step.	Step cycle [s]
Base of support	The average width between the two front or the two hind paws.	Base of support front paws [cm]
Cadence	Number of steps taken per second in a run; it equals: $\frac{n\,u\,m\,b\,e\,r\,o\,f\,s\,t\,e\,p\,s-1}{i\,n\,i\,t\,i\,a\,l\,c\,o\,n\,t\,a\,c\,t\,l\,a\,s\,t\,S\,t\,a\,n\,d-i\,n\,i\,t\,i\,a\,l\,c\,o\,n\,t\,a\,c\,t\,f\,i\,r\,s\,t\,S\,t\,a\,n\,d}$	Cadence [number per second]
Couplings	A temporal relationship between placing two paws on the walkway during one step cycle, i.e., at which moment is the second paw placed in relation to the time of placement of the first one.	Couplings RF → LF [%]
Step sequence	An order of paw placement (footfall pattern). There are 6 footfall patterns (Alternate, Cruciate, Rotate): AA: RF → RH → LF → LH AB: LF → RH → RF → LH CA: RF → LF → RH → LH CB: LF → RF → LH → RH RA: RF → LF → LH → RH RB: LF → RF → RH → LH	Step sequence AA [% of total number of all footfall patterns in a run]
Max contact	A moment in which a paw makes a maximum contact with the glass walkway since the start of a run.	Max contact mean intensity [value of pixels in the range from 0 to 255]
Print positions	A distance between the ipsilateral paws, where the front paw has been placed earlier than the hind paw in the same step cycle.	Print positions right paws [cm]

F, front paw; H, hind; L, left; R, right.

**FIGURE 4:**
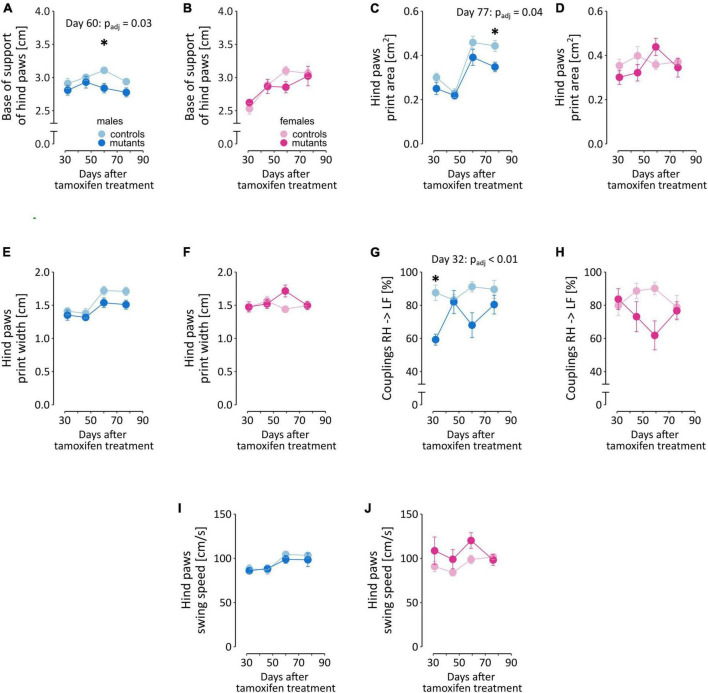
Selected gait parameters in TIF-IA^DATCreERT2^ and control mice. Figures **(A, C, E, G, I)** summarize measurements of the five selected parameters in male mice, and **(B, D, F, H, J)** correspond to the same parameters in female mice. Each point represents the group mean, and error bars are SEM. Female mice are shown in shades of pink, and male mice are shown in blue. Darker hues are TIF-IA^DATCreERT2^ mice, and lighter hues represent the controls. Star (*) indicate significant difference mutants vs. controls at the indicated time point, *t*-tests with Bonferroni correction for multiple tests, test *p* < 0.05. BOS, base of stand; AA, alternate footfall pattern with the paw sequence: RF → RH → LF → LH; RF, right front paw; LF, left front paw.

The TIF-IA^DATCreERT2^ mice differed from controls in several parameters that describe the gait characteristics of hind paws (Figure 4; a full list of ANOVA results is included in Supplementary Table 1). Loss of dopamine neurons affected the gait of males and females to varying degrees. The base of support of hind paws [two-way ANOVA; males, genotype: *F*_(1,17)_ = 4.972, *P* = 0.0395; Figure 4A], their print area [*F*_(1,17)_ = 5.276, *P* = 0.0346; Figure 4C], print width [*F*_(1,17)_ = 4.485, *P* = 0.0492; Figure 4E] and the interval (“couplings”) between the placement of the right hind (RH) and the left front (LF) paws within a step cycle [*F*_(1,16)_ = 16.73, *P* = 0.000855; Figure 4G] were significantly altered in mutant males but not mutant females (Figures 4B, D, F, J, respectively). These 4 parameters were, on average, smaller in value in mutant males than in their control littermates. Conversely, hind paw swing speed was not significantly affected by the mutation in males (Figure 4I), but its effects were significant in females [*F*_(1,17)_ = 5.535, *P* = 0.0309; Figure 4J], with TIF-IA^DATCreERT2^ females having higher swing speed than controls. Significant interactions between time and genotype, the hind paw print width in females only [females: Figure 4F, *F*_(3,51)__=_ 2.826, *P* = 0.0478; Figure 4E for males] and couplings RH → LF were only observed in two cases in males [Figure 4G, *F*_(3,48)_ = 3.161, *P* = 0.0329] but not females (Figure 4H).

Additionally, the time factor was significant in several of the parameters (Supplementary Table 1) that measured hind paw print size [males: days: *F*_(3,51)_ = 44.889, *P* < 0.0001; Figure 4C for males and d for females] and max contact mean intensity of pixels [males: *F*_(3,51)_ = 12.719, *P* < 0.0001; Supplementary Figure 3a for males and Supplementary Figure 3b for females], couplings RF → RH [females: *F*_(3,51)_ = 4.425, *P* = 0.0077; Supplementary Figures 3c, d], alternate step sequence footfall pattern AA [males: *F*_(3,51)_ = 3.068, *P* = 0.036; females: *F*_(3,51)_ = 13.768, *P* < 0.0001; Supplementary Figures 3e, f], swing speed [males: *F*_(3,45)_ = 7.481, *P* = 0.0004; Figures 4I, J], step cycle [males: *F*_(3,45)_ = 7.240, *P* = 0.0005; females: *F*_(3,51)_ = 7.241, *P* = 0.0004; Supplementary Figures 3g, h], cadence [males: *F*_(3,51)_ = 7.054, *P* = 0.0005; females: *F*_(3,51)_ = 4.088, *P* = 0.0112; Supplementary Figures 3i, j], and base of support of the front paws [males: *F*_(3,51)_ = 11.428, *P* < 0.0001; Supplementary Figures 3k, l]. In the cases where no significance between the effect of mutation and time were observed, the changes likely reflect weight gain. Cadence, alternate step sequence footfall pattern AA, hind paw max contact mean intensity of pixels in males and hind paw base of support in females had a tendency to increase over time, while hind paw step cycle tended to decrease, and remaining parameters fluctuated or remained relatively stable between the consecutive test sessions. ANOVA did not detect significant effects in the remaining parameters included in the regression model (Step Sequence CB, Step Sequence RB, Couplings RF → LF and Print Positions Right Paws; Supplementary Table 5).

Taken together, these results show that in the period between 5 and 12 weeks after the mutation was induced, the effects on gait remained at most moderate, and only relatively minor impairments were observed in TIF-IA^DATCreERT2^ mice. The most consistent effects of dopaminergic neuron loss were observed in the parameters related to body weight distribution on the hind paws in male mutants.

### Impact of dopaminergic cell loss on non-motor behaviors in males

**FIGURE 5:**
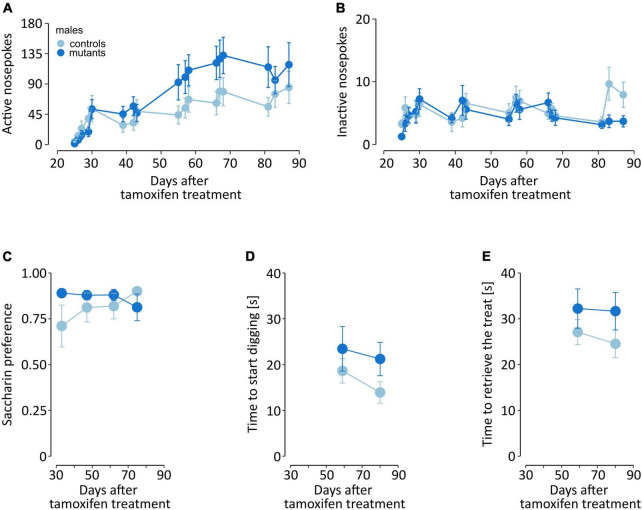
Non-motor behaviors in male mice. The graphs show **(A,B)** instrumental responding in the OSS task, **(C)** two-bottle choice saccharin preference, and **(D,E)** time to find and retrieve a treat in the olfactory acuity test. Points represent the group mean, and error bars are SEM. TIF-IA^DATCreERT2^ mice are shown in dark blue, and the lighter color represents the controls.

In parallel to the gait analysis, we assessed non-motor behaviors potentially relevant to known early PD symptoms. Male TIF-IA^DATCreERT2^ mice were tested at 2- to 3-week intervals for olfactory acuity, saccharin preference and operant sensation seeking (OSS; complete data are presented in Supplementary Table 6). In the OSS task, mice were offered two operands (nose pokes), and the response to one of them was associated with the presentation of a semirandom sequence of blinking lights and tones. C57Bl/6 mice were observed to readily learn to perform responses associated with sensory stimuli, which is interpreted as a sensation-seeking-like behavior (Olsen and Winder, 2009; Rodriguez Parkitna et al., 2013). Importantly, the task did not require food or drink deprivation, which would be a major confounding factor in the case of a model animal with a progressive loss of dopaminergic neurons. We observed that while all mice showed an increase in instrumental responses on the operant associated with the semirandom sensory stimulus over time [Figure 5A, *F*_(16,288)_ = 17.155, *P* < 0.0001], a significant interaction was also observed between time and genotype [*F*_(16,288)_ = 2.844, *P* = 0.0003], but genotype did not have a base effect [*F*_(1,18)_ = 1.452, *P* = 0.244]. On average, the mutants performed 73.9 ± 5.93 active nose pokes per session, while controls only performed 49.6 ± 4.04 nose pokes. Conversely, the number of responses on the “inactive” operant remained low, without genotype [*F*_(1,18)_ = 0.53, *P* = 0.476] or interaction [*F*_(16,288)_ = 1.378, *P* = 0.151] effects, although a significant effect of time was observed [Figure 5B, *F*_(16,288)_ = 2.137, *P* = 0.007]. Thus, partial loss of dopaminergic cell in TIF-IA^DATCreERT2^ males led to increased operant responses over time as a measurement of sensation seeking. At the same time, the male mutants displayed lower counts of inactive responses, similar to those exhibited by the controls. These observations show that the mutants did not present learning or memory deficits and that their behavior was mainly directed toward receiving stimuli.

To assess development of anhedonia-like behavior male mice were tested for saccharin preference every other week, starting 5 weeks after tamoxifen administration. Both mutant and control animals exhibited similar, high preference of saccharin solution vs. water (Figure 5C), the mutation had no significant effect on preference [*F*_(1,15)_ = 1.29, *P* = 0.274, two-way ANOVA].

Next, we tested whether the loss of dopaminergic cells affected their sense of smell. Olfactory acuity was assessed once every three weeks, starting 6 weeks after tamoxifen administration. The first training session is excluded from the analysis. Mutant and control mice exhibited a similar time to start digging at the “correct” corner, i.e., where the cracker was buried underneath the bedding [*F*_(1,18)_ = 2.418, *P* = 0.137, two-way ANOVA; Figure 5D]. Additionally, we also recorded the time in which the mice retrieved the cracker, and found no significant effects of the mutation [*F*_(1,18)_ = 3.121, *P* = 0.094; Figure 5E].

### Probabilistic reversal learning in female TIF-IA^DATCreERT2^ mice

**FIGURE 6:**
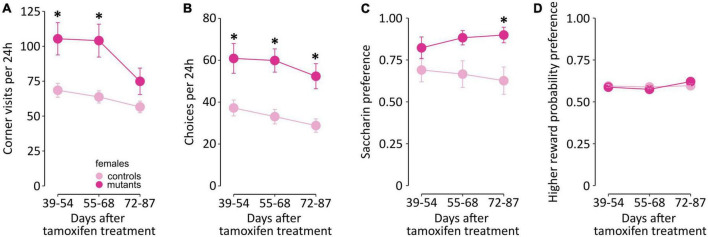
IntelliCage activity in female TIF-IA^DATCreERT2^ and control mice. The graphs show **(A,B)** activity in the cage, **(C)** fraction of choices of the higher reward probability and **(D)** saccharin preference binned over 16-day periods roughly corresponding to the time points from gait testing. Points represent the group mean, and error bars are SEM. Star (*) indicate significant difference mutants vs. controls at the indicated time point, *t*-tests with Bonferroni correction for multiple tests, *p* < 0.05.

Impairment of executive functions was tested in female mice in a probabilistic reversal learning task based on the IntelliCage system. In this version of the task, probabilistic choices are performed in the home cage, without any form of deprivation, and animals remain in a group throughout the procedure. Animals were first habituated to the cage and completed adaptation phase where they were trained to access the corners. The probabilistic choice task started on the 5th week after the mutation was induced. Data were binned in 16-day periods centered around the three latter time points from the gait analysis (Figure 6; data in 24 hours bins are summarized in Supplementary Table 7). Female TIF-IA^DATCreERT2^ mice made more visits per day in the drinking compartments than controls [*F*_(1,19)_ = 10.86, *P* = 0.0044; Figure 6A], especially during the first two periods. Additionally, a significant effect of time [*F*_(2,38)_ = 13.129, *P* < 0.001] and a significant interaction of time and genotype [*F*_(2,38)_ = 3.954, *P* = 0.028] were observed. The number of choices (visits longer than 2 s only to the compartments offering saccharin) followed the same course, with a significant increase in mutants across all time periods [*F*_(1,19)_ = 16.34, *P* < 0.001; Figure 6B] and a genotype-independent effect of time [*F*_(2,38)_ = 6.252, *P* = 0.005], but no significant interaction [F_(2,38)_ = 0.281, *P* = 0.757]. Genotype did not affect the preference of the choice with a higher probability of accessing reward [*F*_(1,19)_ = 0.007, *P* = 0.936; Figure 6C]. Both control and mutant mice showed consistent preference for the “90%” corner [59.3 ± 0.594% (SEM) and 59.4 ± 0.825% of visits in the compartments offering saccharin, respectively]. However, significant effects of time period [*F*_(2,38)_ = 5.262, *P* = 0.009] and the interaction of the two factors [*F*_(2,38)_ = 3.688, *P* = 0.034] were observed. Interestingly, TIF-IA^DATCreERT2^ mice had a significantly higher preference for saccharin than controls [i.e., the fraction of licks on saccharin bottles, Figure 6D; *F*_(1,19)_ = 4.727, *P* = 0.043], with an average fraction of drinking saccharin at 0.661 ± 0.0436% (SEM) in controls and 0.868 ± 0.0297 in mutants, and an interaction effect on the preference over time [*F*_(2,38)_ = 3.290, *P* = 0.048] was observed. Thus, female mutant mice performed a greater number of responses in the task and had higher saccharin preference but were not different in the fraction of choices of the higher probability of reward.

### Correlations between gait parameters and non-motor behaviors tested

**FIGURE 7:**
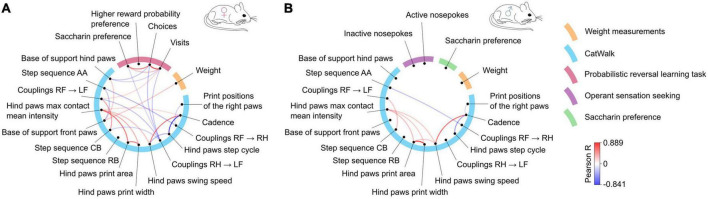
Correlations between gait parameters and non-motor behaviors in **(A)** females and **(B)** males. The blue semicircle groups motor parameters, each of them represented by a dot and labeled outside the circle. The remaining colors correspond to the non-motor parameters. Lines connecting the dots represent significant (R values corresponding to *p* < 0.05) positive (red) or negative (blue) correlations according to the scale shown on the right. The correlation analysis was performed jointly for mutants and controls to determine whether motor and non-motor parameters change overtime in a similar manner.

Finally, we have analyzed the complete data set to assess if there were specific correlations between the development of motor and non-motor symptoms. Comparisons of the parameters measured were assessed separately in male and female mice (Figures 7A, B, respectively, and Supplementary Table 8 with complete correlation matrices). Significant positive correlations were observed between motor parameters associated with hind paw print size (area and width; Pearson R: males: *R* = 0.89, females: *R* = 0.75) or the hind paw swing speed and cadence (males: *R* = 0.76, females: *R* = 0.73), and significant negative correlations existed between cadence and hind paw step cycle (males: *R* = −0.63, females: *R* = −0.84) or step sequence AA and coupling of steps between the right paws RF to RH (males: *R* = −0.51, females: *R* = −0.55). These pairs of parameters are linked to related gait parameters and, accordingly, were observed very consistently in both male and female mice. In female mice, several significant correlations were observed between motor and non-motor measures. The base of support for the front paws positively correlated with weight (*R* = 0.48), which likely reflects the differences in size of the animals (and the increase in size during the experiment). The non-motor behaviors in females showed strong correlations in closely related measures (e.g., positive correlation between number of visits and number of choices in PRL task, *R* = 0.87) but also had significant correlations with the gait parameters. The two measures related to the number of corner visits during the probabilistic reversal task had a negative correlation with the base of support for the hind paws (visits: *R* = −0.41, choices: *R* = −0.47) and coupling of steps (RH to LF; visits: *R* = −0.47, choices: *R* = −0.5). Similarly, saccharin preference was also significantly correlated with the coupling parameter (*R* = −0.42), as well as with the number of visits (*R* = 0.51) and number of choices (*R* = 0.72). In contrast to these observations in females, saccharin preference, weight and the number of active or inactive nose pokes in the instrumental task were not significantly correlated with gait performance in male mice (Supplementary Table 8). Moreover, the number of active and inactive responses performed in the operant sensation seeking task were not closely intertwined (*R* = 0.32, *P* > 0.05), which implies that the high number of active nose pokes did not depend on the total number of instrumental responses.

Taken together, two observations emerged in the analysis. First, the results from males and females differed, specifically, the lack of correlation between motor and non-motor parameters in males. Second, the distance between hind paws, which was the parameter with the strongest genotype effect, also appeared to have the highest correlation to non-motor parameter measures, although as noted, significant effects were observed only in females.

## Discussion

We find that the progressive loss of dopaminergic neurons in mice is associated with robustly increased instrumental responding but normal learning of action-outcome contingencies and ability to assess the probability of an action being rewarded. Moreover, this loss was not associated with a decrease in preference for sweet taste. Increased instrumental responding correlated with early changes in gait (the base of stand and step coupling), although these effects were observed only in female mice. These observations were derived from early stages of degeneration in a model of slowly progressing loss of dopaminergic neurons and include the effect of sex, and thus provide valuable insight into the role of altered dopamine signaling in early PD symptoms.

Analysis of gait in TIF-IA^DATCreERT2^ mice revealed only minor alterations up to 12 weeks after inducing the mutation. Previously, the progression of neurodegeneration in the substantia nigra pars compacta was assessed as under 20% at 7 weeks after tamoxifen treatment and 50% at 10 weeks, with loss of dopamine in the striatum reaching 80% at 10 weeks (Kreiner et al., 2019). In humans, the emergence of motor symptoms coincides with loss of dopaminergic neurons in the substantia nigra exceeding ∼30% and a decrease in dopamine concentration in the caudate of ∼70% (Cheng et al., 2010). We confirmed that only traces of TH immunoreactivity remained in the striatum of TIF-IA^DATCreERT2^ mice killed at 18 weeks after tamoxifen induction. Thus, the observed changes in gait were milder than expected, especially at 11 weeks after mutation induction. It should be noted though, that previous reports using the TIF-IA^DATCreERT2^ mouse model have only used rotarod to measure motor performance, which is not directly comparable to analysis of gait, as seen in studies of different brain dysfunctions (Henry et al., 2020; Jacquez et al., 2021; Wang et al., 2023). Moreover, we have applied rigorous control for the number of parameter tested when assessing the significance of observed changes, which may have limited the power to detect milder effects.

Significant effects of the mutation were observed primarily in parameters related to the distance between the hind paws and timing of placing two paws (couplings RH → LF), although we note that the results were not fully consistent between male and female mice. The results are in partial agreement with those of previous studies that used similar methodology (Kappos et al., 2017; Frohlich et al., 2018; Timotius et al., 2019; Pitzer et al., 2021). Direct comparison of the parameters is confounded by differences in parameter reduction approaches and, most importantly, the stage of dopaminergic degradation in the animals tested. In a study where transgenic rats overexpressing human α-synuclein were tested for their performance in the Catwalk, the earliest (at the age of 10 to 12 weeks) observed changes in gait were related to hind paw base of stand and hind body speed (Timotius et al., 2019), which is consistent with our results. Furthermore, the effect of the mutation on weight gain may affect gait and thus represent a confounding factor, although we only observed a correlation between weight and the distance between front paws in females. Moreover, gait impairments in PD patients were found to be only partly remediated by dopaminergic treatment (Sethi, 2008; Mirelman et al., 2019; Wilson et al., 2020), which may also explain the small degree of gait dysfunction observed in TIF-IA^DATCreERT2^ mice. It should also be noted that disruptions in cholinergic system are also implicated in PD motor dysfunction, including gait and postural dysfunctions (Rochester et al., 2012; Müller and Bohnen, 2013; Nazmuddin et al., 2021), therefore, the limited effects on gait observed in TIF-IADAT^CreERT2^ mice could be due to lack of cholinergic deficits. Altogether, we found only relatively minor changes in the gait of TIF-IA^DATCreERT2^ mice, which confirms the limited degeneration of dopaminergic neurons and their impact on gait and excludes movement impairment as a factor that could have affected the testing of non-motor behaviors.

Loss of olfactory acuity was reported as a frequent prodromal PD symptom (Postuma et al., 2012). Dopamine signaling plays a major role in the rodent olfactory system (Pignatelli et al., 2005; Escanilla et al., 2009; Zhou et al., 2022), and thus, a change in the abundance of dopaminergic neurons could be expected to affect the sense of smell in mice. PD patients have been observed to have an increased number of dopaminergic cells in the olfactory bulb (Huisman et al., 2004; Mundiñano et al., 2011), and a direct lesion of dopaminergic neurons in the olfactory bulb prevents the development of hyposmia in 6-hydroxydopamine-treated rats (Ilkiw et al., 2019). However, as reported previously, the mutation in TIF-IA^DATCreERT2^ primarily affects midbrain dopaminergic neurons without appreciable effects on the olfactory system (Papathanou et al., 2019). Accordingly, we observed no change in TH-positive cell abundance in the olfactory bulb of TIF-IA^DATCreERT2^ mice. Thus, the lack of genotype effects on olfactory acuity in the task requiring a buried treat is consistent with the properties of the Cre transgene in the mutant strain, since DAT expression is almost exclusive to midbrain dopaminergic neurons (Cerruti et al., 1993; Ciliax et al., 1999), and, therefore, olfactory dopaminergic cells were probably not affected directly by mutation. It should also be noted that cholinergic dysfunction has also been shown to contribute to hyposmia in PD (Bohnen et al., 2010; Perez-Lloret and Barrantes, 2016). Even though, we cannot fully dismiss the possibility that the loss of midbrain dopaminergic cells had an impact on olfactory neurons, as it has been shown in another study (Zhang et al., 2019).

We found no decrease in saccharin preference in mutant mice and thus no anhedonia-like behavior that could be interpreted as a symptom of a depression-like phenotype (Papp et al., 1991). The significant increase in preference observed in female mutant mice stems from the larger number of choices performed, and we believe that the volume of sweetened water intake itself is unlikely to represent an increase in reward-seeking, since it was not replicated in male animals. Previously, we reported that mice lacking NMDA receptors in dopaminergic neurons (the NR1^DATCreERT2^ strain) had a phenotype resembling some depression symptoms; however, these animals also had a normal preference for saccharin (Jastrzębska et al., 2016). Therefore, the absence of anhedonia alone is not sufficient to rule out other depressive-like symptoms. Furthermore, it should be noted that affective symptoms observed in PD patients could also stem from degeneration of non-dopaminergic systems (Gallagher and Schrag, 2012; Castrioto et al., 2016), while the animal model used here targets specifically only a subpopulation of dopaminergic neurons.

The most consistent, non-motor effect of the mutation was increased activity in instrumental tasks. A higher number of responses was observed in males in the OSS task and in females in the IntelliCage-based probabilistic reversal learning (PRL) task. As noted, this change was not associated with decreased accuracy, and responses on the inactive lever in the OSS and preference for higher reward probability in the PRL task were not affected by genotype. Therefore, TIF-IA^DATCreERT2^ mice were not impaired in instrumental learning. We and others have previously reported that performance in the OSS task is highly sensitive to the loss of metabotropic glutamate receptors (including a selective mutation in neurons expressing dopamine D1 receptors) (Olsen et al., 2010; Rodriguez Parkitna et al., 2013) and to treatment with opioid receptor antagonists (Sikora et al., 2019). Moreover, the activity of dopamine D2 receptors was previously indicated as a key regulator of sensation seeking (Norbury et al., 2015; Norbury and Husain, 2015); thus, we anticipated a change in the behavior of TIF-IA^DATCreERT2^ mice in the OSS. Nevertheless, the observed increase, rather than decrease, is counterintuitive, even more so since PD patients may display lower sensation seeking (Evans et al., 2006). The increase in responding could be speculated to reflect compensatory mechanisms that emerge after a partial loss of dopaminergic neurons (Brotchie and Fitzer-Attas, 2009), and this notion would be consistent with the observed time course—the strongest effect was apparent 7 to 9 weeks after the induction of the mutation, and responding returned to control levels at the 11-week mark.

The lack of genotype effect on worsened performance (i.e., lower frequency of obtaining rewards or fewer rewards obtained) in the PRL task strongly implies normal executive functions in TIF-IA^DATCreERT2^ up to 11 weeks after inducing the mutation. In humans, PD reportedly causes a shift in sensitivity toward negative outcomes in the PRL task, which was influenced by L-DOPA treatment (Frank et al., 2004; Cools et al., 2006), and possibly a general modest impairment in the PRL task (Grogan et al., 2017; Meder et al., 2019). However, these observations are subject to continuing discussion, and the higher sensitivity to negative outcomes is especially debated (Meder et al., 2019). The reports were consistent in showing altered and likely impaired reinforcement learning in persons with PD, and impaired dopamine signaling was indicated as plausible cause. Due to the strict time constraints and predetermined structure of the PRL tasks applied in humans, an increase in choices like that observed here could not be detected. Moreover, we note that the intervals between choices in the task presented here were usually between 1 and 20 minutes, compared to seconds in typical human PRL tasks. Thus, while the data reported here show no impairment in executive functions in mice during the early stages of dopaminergic neuron degeneration, the increase in instrumental responding certainly warrants investigation in humans, which would necessitate a redesign of the usually applied PRL tasks. This idea is supported by the analysis of correlations between observed motor and non-motor behaviors. The number of choices and visits performed by female TIF-IA^DATCreERT2^ mice in the PRL task was negatively correlated with hind paw swing speed and base of support. The hind paw swing speed could be considered to have the highest similarity to bradykinesia; thus, the negative correlation to instrumental choices suggests that it may have a common underlying mechanism. Nevertheless, we do note the caveat that the swing speed data have high variability, and instrumental responses in the OSS in males were not significantly correlated with gait parameters. Taken together, we find no indication of altered reinforcement learning in animals with partial loss of dopaminergic neurons, which probably supports the general notion that cognitive impairments observed in PD in humans are attributed mainly to degeneration or dysfunction of non-dopaminergic neurons (Halliday et al., 2014), mostly cholinergic (Perry et al., 1985; Perez-Lloret and Barrantes, 2016; Albin et al., 2022) and noradrenergic (Riekkinen et al., 1998; Loued-Khenissi and Preuschoff, 2015).

We should note that the interpretation of the results is to an extent limited by two important caveats. First is the difference in behavioral tests performed on male and female animals. This was necessitated by technical limitations of the procedures, as the presence or scent of the opposite sex affects behavior and it was impossible to fit them in the same schedule. Additionally, aggressive behaviors among male mice housed in larger (over 10 individuals) groups limit the possibility to observe their behavior over long periods in IntelliCages. Thus, while the difference in the experimental schedules makes it impossible to extract the effect of sex, we note that the effect of the mutation on the number of choices or preference or lack of the effect of sweet taste was observed under two different paradigms. The similarity in behavior in operant tasks of both male and female TIF-IA^DATCreERT2^ mice (i.e., increased activity in the tests in comparison to controls) is in line with previous finding of no sex-related difference in cognitive state in *de novo* PD patients with 4-year follow up (Bayram et al., 2020). Nevertheless, no significant effect of sex on PD-like phenotypes in mice contrasts with some other observations from studies on male and female PD patients cohorts (Picillo et al., 2016; Perrin et al., 2017; Li et al., 2023). Some reports indicate that male PD patients experience greater decline of motor and non-motor function, though no sex-dependent differences in progression rate were found (Abraham et al., 2019, 2023; Picillo et al., 2022; Li et al., 2023).

## Conclusion

In summary, we found relatively few changes in behavior in the early stages of dopaminergic neuron degeneration in a genetically modified mouse strain, and the largest effect of genotype was observed in operant behavior. These results do not support a contribution of a partial loss of dopaminergic neurons to impaired executive functions in the prodromal phases of PD; however, they also suggest that increased instrumental responding merits investigation as a potential marker of early stages of disease development.

## Data availability statement

The datasets presented in this study and code used in the analysis can be found in online repositories. The R scripts used in the statistical analyses are available at the GitHub page of the experiment (https://github.com/annaradli/tif-pd-behavior). Raw behavioral data can be downloaded from the Zenodo webpage (https://zenodo.org/records/10104172).

## Ethics statement

The animal study was approved by 2nd Local Institutional Animal Care and Use Committee in Kraków, Poland. The study was conducted in accordance with the local legislation and institutional requirements.

## Author contributions

AR-B: Conceptualization, Formal analysis, Investigation, Visualization, Writing – original draft. JJ: Formal analysis, Software, Writing – review & editing. ML: Formal analysis, Software, Visualization, Writing – review & editing. ŁS: Investigation, Writing – review & editing. ZH: Investigation, Methodology, Writing – review & editing. MB: Resources, Writing – review & editing. JB: Formal analysis, Investigation, Visualization, Writing – review & editing. JoP: Conceptualization, Writing – review & editing. GK: Conceptualization, Resources, Writing – review & editing. DW: Conceptualization, Formal analysis, Writing – review & editing. JRP: Conceptualization, Formal analysis, Supervision, Visualization, Writing – original draft.
