# Supplementary material for: Non-motor symptoms associated with progressive loss of dopaminergic neurons in a mouse model of Parkinson’s disease
Source: Front Neurosci. 2024 Apr 30;18:1375265. doi: 10.3389/fnins.2024.1375265 (PMC11091341; doi:10.3389/fnins.2024.1375265)
Supplement: Supplementary file 9 [file Data_Sheet_1.docx]

**Supplementary material captions**

Supplementary Figure 1. Tyrosine hydroxylase (TH) immunofluorescence signal in coronal sections of substantia nigra pars compacta (SNc) and ventral tegmental area (VTA) in control (left) and mutant (right) TIF-IA^DATCreERT2^ male mice at 14^th^ week after induction of mutation via tamoxifen administration. The figure shows corresponding sections from each group of anterior parts of the structures (at the top) to posterior sections (at the bottom). White bar corresponds to 1 mm.

Supplementary Figure 2. Tyrosine hydroxylase (TH)-positive cell counts in three control and three mutant TIF-IA^DATCreERT2^ male mice at 14^th^ week after induction of mutation via tamoxifen administration in substantia nigra (SN; a) and ventral tegmental area (VTA; b). Cell counts are expressed as mean percentage of control number of TH-positive cells. Error bars indicate SEM.

Supplementary Figure 3. CatWalk parameters identified by the linear regression model that were most closely related to genotype (Figure 4 continued).

Supplementary Table 1. Summary of two-way ANOVAs of all behavioral tests and weight measurements for males and females.

Supplementary Table 2. CatWalk complete raw dataset.

Supplementary Table 3. Correlation coefficients of CatWalk parameters between the left and right paws.

Supplementary Table 4. CatWalk parameters used in linear regression model reduction of data.

Supplementary Table 5. Results of linear regression analysis of CatWalk parameters using the most efficient model.

Supplementary Table 6. Operant sensation-seeking data.

Supplementary Table 7. IntelliCage data summarized in bins.

Supplementary Table 8. Correlation matrices for assessing the relationship between motor and non-motor functions.
