# Supplementary figures and images for: Non-motor symptoms associated with progressive loss of dopaminergic neurons in a mouse model of Parkinson’s disease
Source: Front Neurosci. 2024 Apr 30;18:1375265. doi: 10.3389/fnins.2024.1375265 (PMC11091341; doi:10.3389/fnins.2024.1375265)

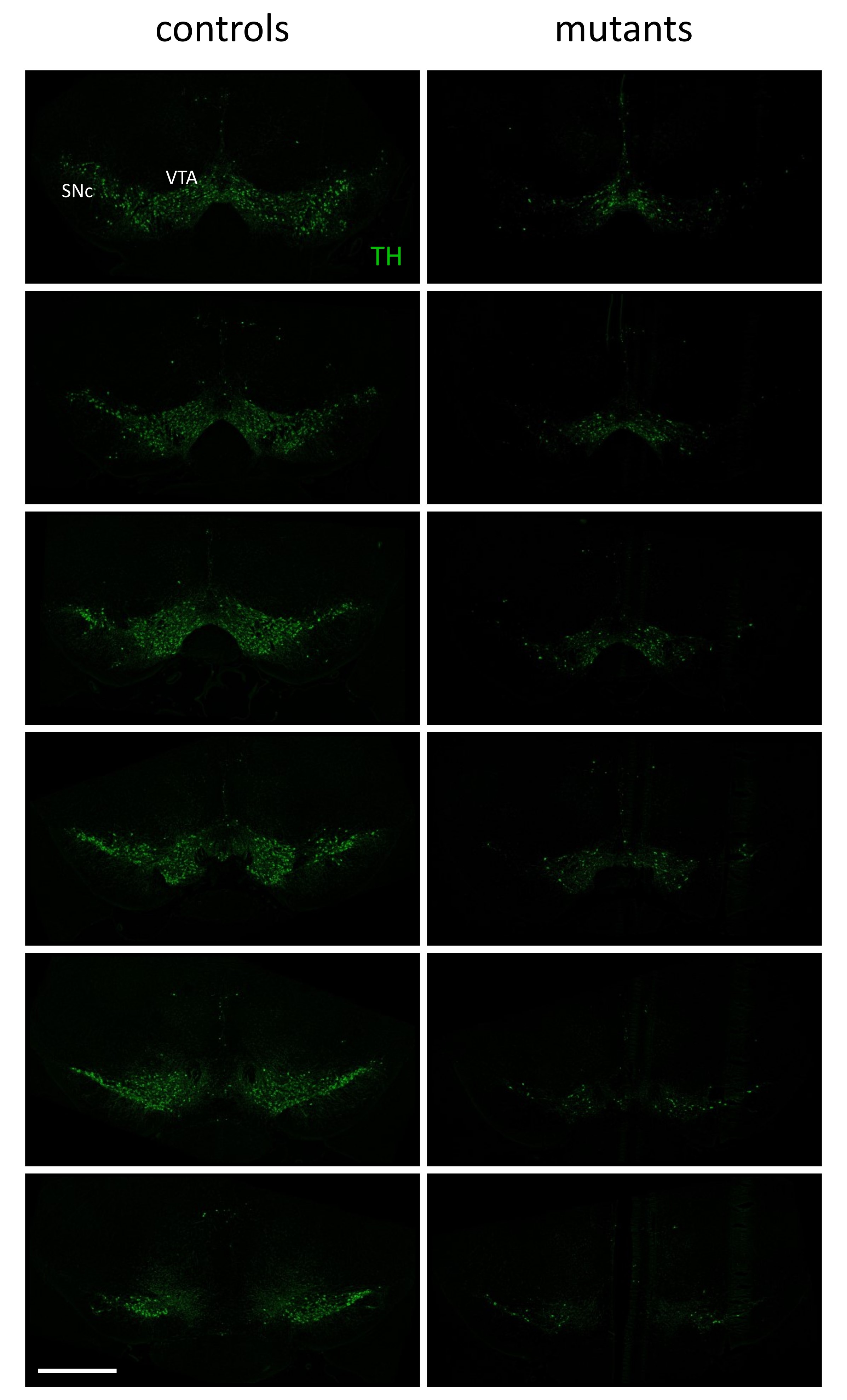

Supplement: Supplementary file 10 [file Image_1.JPEG]

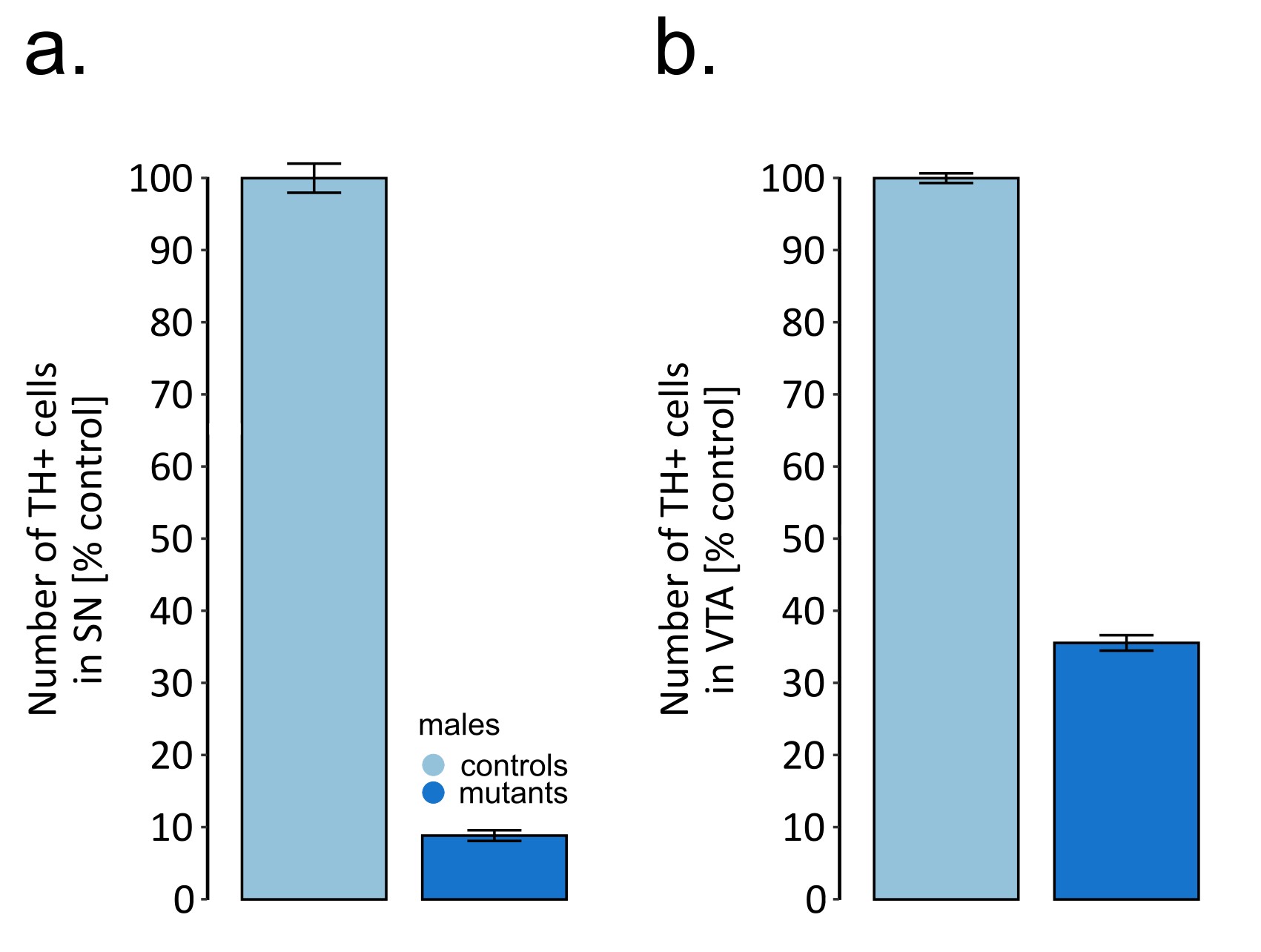

Supplement: Supplementary file 11 [file Image_2.JPEG]

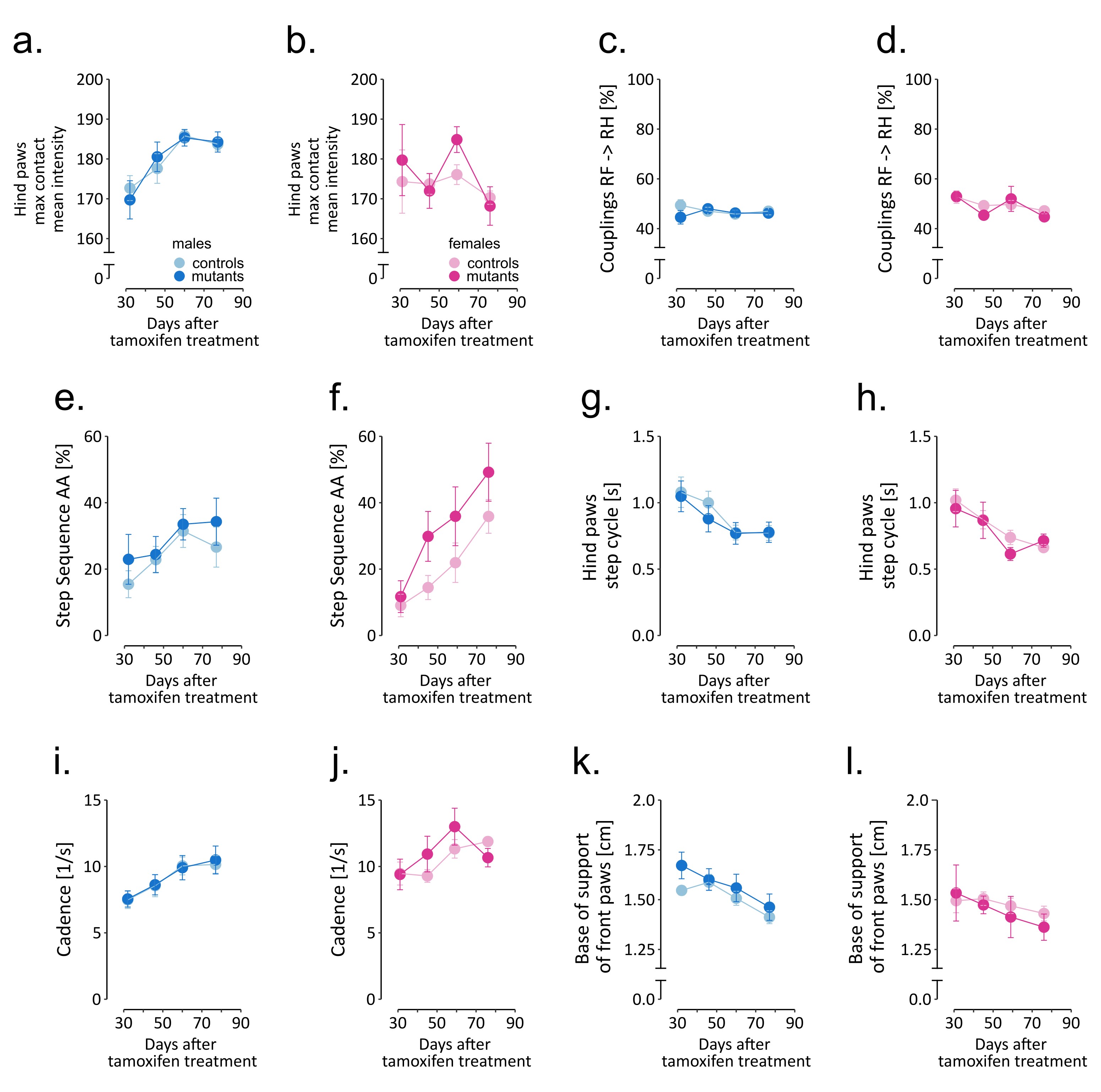

Supplement: Supplementary file 12 [file Image_3.JPEG]
